# Biliary tract microbes and common bile duct stones: current status and prospects

**DOI:** 10.3389/fmicb.2026.1818256

**Published:** 2026-06-16

**Authors:** Chen Mengjia, Wang Bujiang, Chen Honghui, Hu Qiying, Song Haojun

**Affiliations:** 1 Health Science Center, Ningbo University, Ningbo, China; 2 Department of Gastroenterology, The First Affiliated Hospital of Ningbo University, Ningbo, China; 3 Ningbo Key Laboratory of Translational Medicine Research on Gastroenterology and Hepatology, The First Affiliated Hospital of Ningbo University, Ningbo, China

**Keywords:** biliary microbiota, common bile duct stones, diagnosis, microbial dysbiosis, pathogenesis

## Abstract

Common bile duct stones is a common digestive system disease, and about 5%−30% of patients with cholelithiasis are complicated with common bile duct stones. It poses significant challenges to clinical diagnosis and treatment. Although its occurrence is related to traditional factors such as abnormal bile composition and biliary dynamics disorders, the exact pathogenesis has not been fully clarified. In recent years, with the rapid development of high-throughput sequencing and metagenomics and other microbiome technologies, researchers have begun to pay attention to the role of biliary microbiota in the formation of common bile duct stones. More and more evidence indicates that the biliary tract microbes may has been associated with the occurrence and development of stones. This review firstly examines the literature implicating between biliary microorganisms and different types of common bile duct stones. We discuss the various mechanisms of action of biliary tract microorganisms in the occurrence of common bile duct stones. We also evaluated the specific value of microbial markers for diagnostic typing and prediction of recurrence.

## Graphical Abstract

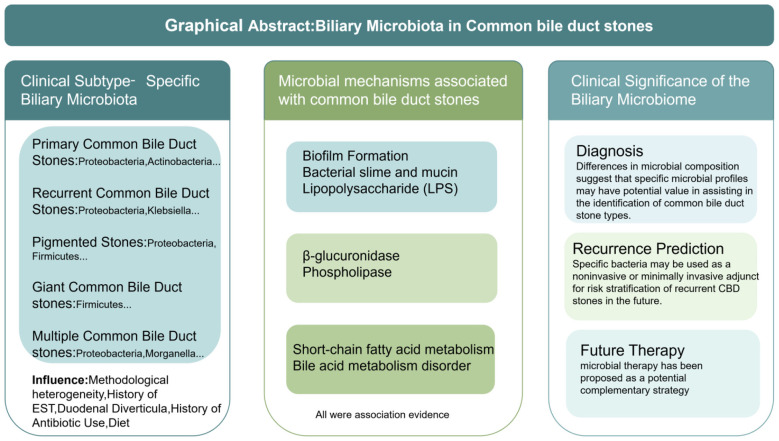

## Introduction

Cholelithiasis is a globally common digestive system disease. Common bile duct stones, a clinically important type, is found in approximately 5%−30% of patients with cholelithiasis (Wu et al., 2021). It has been established that they are the direct cause of biliary obstruction, and that this can result in serious complications, including acute cholangitis and pancreatitis (Disario et al., 2004). The mortality rate associated with these complications is reported to be as high as 10%−30% (Chang et al., 2018; Cheng et al., 2016; Shabanzadeh et al., 2016). It represents a considerable strain on the social and medical systems. Studies have shown that the prevalence of cholesterol stones is higher in Western countries. In Asia, the incidence of common bile duct stones may be increased because of the greater prevalence of pigment stones (Calomino et al., 2025; Velanovich et al., 2006).

Study revealed that the supersaturation of cholesterol and an imbalance of bile acids can promote the precipitation of cholesterol crystals (Sun et al., 2022). Study have shown that bile pigment stones is linked to anatomical abnormalities and problems with the sphincter of Oddi (Wu et al., 2007). The development of common bile duct stones was speculated to be related to classic factors such as changes in bile composition, an imbalance in cholesterol homeostasis and dysfunction of biliary tract dynamics. However, although

these mechanisms have been extensively studied, the exact pathogenesis of common bile duct stones has not been fully elucidated. In recent years, with the breakthrough of microbe group technology, the researchers report in the biliary system there is a stable microbial community. Studies proposed that that the occurrence and development of choledocholithiasis may be related to a variety of putative pathways, such as abnormal bile acid metabolism (Dai et al., 2023), biofilm formation (Xiao et al., 2024), bacterial enzyme secretion (Trotman, 1991), and other mechanisms.

Despite the growing research on biliary microbiota, the research on the mechanism of common bile duct stones is still in its infancy, and the causal mechanism and clinical transformation path through which the microbial community affects the occurrence and recurrence of common bile duct stones are not clear. Existing reviews mostly focus on gallstones, and rarely discuss the association between biliary microbiota and different subtypes of common bile duct stones.

Accordingly, this review aims to move beyond a mere summary of reported associations between biliary microbiota and common bile duct stones to provide a critical and comprehensive analysis of their intricate relationship. This review is the first to systematically summarize the specific flora spectrum of common bile duct stones in different clinical subtypes (including primary, recurrent, pigmented, giant, and multiple stones).We systematically examine the potential roles of biliary microorganisms in the formation, recurrence, and clinical management of common bile duct stones, with a specific focus on: (1) characterizing subtype-specific microbial signatures across distinct clinical subtypes of common bile duct stones, including primary, recurrent, pigmented, giant, and multiple stones; (2) critically evaluating the strength of evidence distinguishing causality from correlation, while addressing key methodological challenges and confounding factors; (3) elucidating the mechanistic pathways driving stone formation; and (4) accelerating to clinical translation, including diagnostic biomarkers, recurrence prediction models, and microbiome-based therapeutic strategies.

## Composition and characteristics of biliary tract microbiota

The microbiota is important for maintaining the health of the body and is closely related to the biliary system. Bile was considered sterile in the past, but in the 20th century, Maluenda et al. reported the presence of several types of bacteria in bile from common bile duct stones, especially Gram-negative aerobic bacteria such as *Escherichia coli* and *Klebsiella* (Maluenda et al., 1989). With the development of sequencing technology, studies based on 16S rRNA sequencing and metagenomics have confirmed that the composition of the biliary microbiota is richer than previously thought and that a distinct microbial community resides within the biliary tract. On the basis of existing studies, the core phyla constituting the biliary microbiota are Proteobacteria, Firmicutes, Bacteroidetes, and Actinobacteria (Azimirad et al., 2023; Chen et al., 2019). The dominant phyla were Proteobacteria and Firmicutes (Han et al., 2021). At the genus level, different clinical types of stones have specific microbiota profiles: *Pseudomonas* and *Escherichia–Shigella* were the predominant genera in primary common bile duct stones (Lyu et al., 2021), recurrent stones have significant enrichment of *Klebsiella* (Tan et al., 2022) and *Escherichia* (Ye et al., 2020), pigmented stones are characterized by increased *Enterococcus* (Kim et al., 2021) and *Escherichia Shigella* abundance (Zhang et al., 2025), *Morganella* is seen as a specific marker for multiple stones (Xia et al., 2025) and the proportion of *Enterococcus* was the highest in the giant common bile duct stones (Dai et al., 2023).

**Figure 1:**
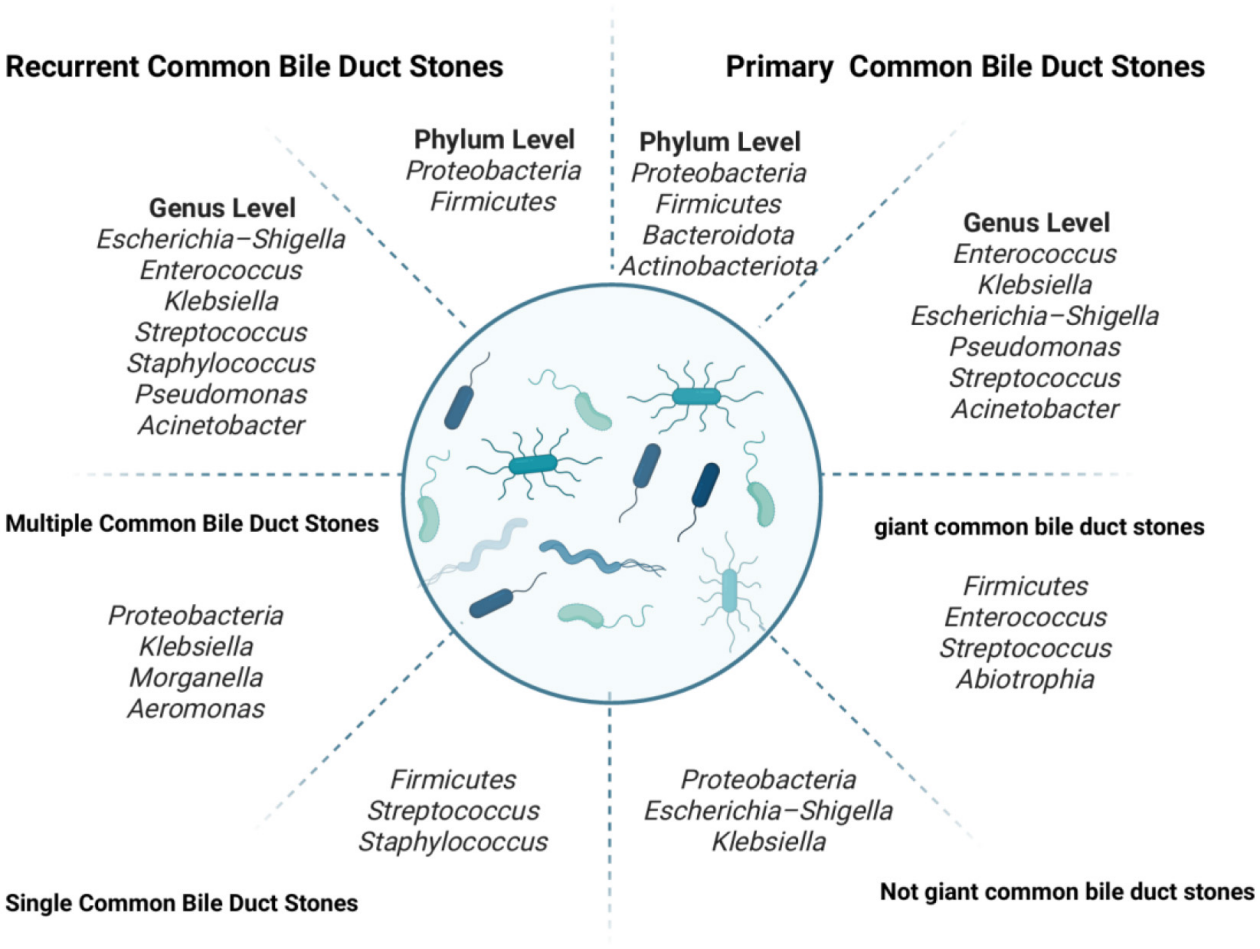
Biliary microbiota composition in recurrent, multiple, giant, non-giant, and single common bile duct stones. Phylum- and genus-level taxa are shown. Proteobacteria and Firmicutes are dominant across most subtypes, with subtype-specific enrichments (e.g., *Morganella* in multiple stones, *Enterococcus* in giant stones). Findings are observational and require prospective validation.

The biliary tract and duodenal microbiomes have several similarities and differences. Studies have shown that the biliary tract and duodenal microflora have the same core microflora structure (Kim et al., 2021; Lee et al., 2022). Han et al. reported the similarity between the microbiome of duodenal fluid and bile in patients with common bile duct stones and proposed that duodenal-bile reflux and common bile duct stones may be associated at the microbiome level (Han et al., 2021). Liu et al. (2024) reported that the duodenal microbiota may be the main source of biliary microbiota (Liu et al., 2024). These findings support the retrograde migration of intestinal bacteria *via* the duodenal papilla as a major source of biliary microbiota. In general, the biliary tract microbes in patients with common bile duct stones are likely derived from intestinal microbes, but the microecology of the biliary tract and duodenum is complex. A recent study has shown that the microbial diversity of Primary Common Bile Duct Stone Patients with Juxtapapillary Duodenal Diverticula is different from that of simple common bile duct stones (Figure 1; Wang et al., 2025).

## Clinical subtype-specific biliary microbiota in common bile duct stones

The biliary microbial compositions of patients with common bile duct stones show systematic differences due to the origins, compositions, and morphological characteristics of the stones. Many studies have indicated that biliary microbiota is closely related to common bile duct stones. The composition of the biliary microbiota in patients with primary and recurrent common bile duct stones is similar at the phylum level, with Proteobacteria and Firmicutes being the absolute dominant flora (Azimirad et al., 2023; Chen et al., 2019; Han et al., 2021). However, significant differences in the abundance, diversity and types of bacterial genera were detected between the different groups.

### Primary common bile duct stones

One study found that short-chain fatty acid (SCFA) -producing bacteria were significantly reduced in the biliary microbiota of primary common bile duct stones and *Pseudomonas* was significantly increased (Lyu et al., 2021). Another study of primary common bile duct stones found that the abundance of *Pseudomonas aeruginosa* was not significant, which may be related to the different location of bile samples. But they found that *Pseudomonas aeruginosa* may be involved in biofilm formation and the abundance of *Enterococci* increased (Xiao et al., 2024). Liu et al. (2023) focused on Actinobacteria. They found that the abundance of Actinobacteria in the stone group was lower, which was consistent with the results of most studies. In addition, they also found that the abundance of Actinobacteria was negatively correlated with the risk of occurrence and recurrence of primary common bile duct stones (Liu et al., 2023). According to existing researches, the dominant phyla of biliary microbiota in patients with primary common bile duct stones are relatively stable, but the key genera are different. The decreased abundance of Actinobacteria was a relatively consistent finding, while the increased abundance of *Pseudomonas* and *Enterococci* may be involved in the information of stones. However, there are still inconsistencies in the abundance changes of specific bacterial genera among different studies, which may be related to sample source, patient population and experimental method.

### Recurrent common bile duct stones

The most striking features of patients with recurrent common bile duct stones are reduced microbial diversity and enrichment of specific pathogenic genera. At the phylum level, the relative abundance of Proteobacteria significantly increased in patients with recurrent stones (Tan et al., 2022; Ye et al., 2020). Although the dominant genera reported in different studies differ, the enrichment of *Escherichia, Klebsiella* (Tan et al., 2022) and *Enterococcus* is a feature of patients with recurrent common bile duct stones (Liu et al., 2024). Study have found that in patients with Audi sphincter relaxation, the abundance of *Clostridium* in the bile of those with recurrent stones is significantly increased. This suggests that *Clostridium* may be associated with stone recurrence (Zhang et al., 2020). There are many factors for the recurrence of common bile duct stones. Besides some known factors, the role of the microbiota in the recurrence of common bile duct stones is gradually being recognized.

### Pigmented stones

The formation of pigmented common bile duct stones, especially brown stones, is most probably associated with bacterial infection. Early bacterial culture studies revealed that up to 80.5% of brown pigmented common bile duct stones were positive for bacteria, among which Enterococcus was the most common microorganism (Leung et al., 1989). A study revealed that the biliary microbiota is dominated by Proteobacteria, Firmicutes, and Bacteroidetes and that the abundance of *Enterococcus* is significantly enriched at the genus level (Kim et al., 2021). However, Zhang et al. (2025) showed that *Actinomyces, Streptococcus*, and *Achromobacter* were more enriched in the stones, while *Enterococcus* was detected but not the most prominent (Zhang et al., 2025). This discrepancy may be related to the differences in sample types, control Settings and populations, suggesting that the consistency between different studies needs to be verified by larger multicenter studies.

### Special types

With respect to special types such as giant and multiple common bile duct stones, preliminary studies have revealed unique microbial profiles. In patients with giant common bile duct stones, the abundance of Firmicutes is significantly greater, and its unique composition at the genus level includes *Enterococcus, Citrobacter, Lactobacillus*, etc. (Dai et al., 2023). In patients with multiple common bile duct stones, compared with those with single stones, the abundance of Proteobacteria at the phylum level significantly increased, whereas at the genus level, specific enrichment of *Klebsiella, Morganella* and *Aeromonas* was detected. Notably, *Morganella* was specifically found to be present in the multiple stone group, suggesting that it may serve as a potential specific microbial marker for patients with this type of stone (Xia et al., 2025). These studies revealed the correlation between biliary microorganisms and common bile duct stones from different perspectives and provide a basis for further exploration of the underlying mechanism.

To provide a systematic overview of the biliary microbiota landscape in common bile duct stones, we summarized representative studies in Table 1. The table is organized by disease subtypes (e.g., primary, recurrent, pigmented, giant, and multiple stones) and includes information on control groups, sample types, sequencing methods, and reported microbial compositions.

**Table 1:** Study of the biliary microbiota in common bile duct stones.

Disease types	Contral group	Sample	Method	Microbiological composition	References
Common bile duct stones	Hepatobiliary disorders without CBD stones	Bile	16S rDNA	Phyla: Proteobacteria, Firmicutes, Actinobacteria, and Bacteroidetes Genus: *Cronobacter, Pseudomonas, Enterococcus, Achromobacter, Klebsiella, and Enterobacter*	Azimirad et al. (2023)
	No control group	Bile + duodenal fluid	16S rDNA	Phyla: Proteobacteria, Firmicutes Genus: *Escherichia-Shigella, Fusobacterium, and Enterococcus*	Han et al. (2021)
	Cholangiocarcinoma	Bile	16S rDNA	Phyla: Proteobacteria, Firmicutes, Bacteroidetes, and Actinobacteria Genus: *Escherichia, Halomonas, Klebsiella, Streptococcus, and Enterococcus*	Chen et al. (2019)
	Cholecystolithiasis	Bile	16S rDNA	Phyla: Proteobacteria, Firmicutes, Fusobacteria, Bacteroidetes, and Actinobacteria Genus: *Ralstonia, Bacteroides, Prevotella, and Enterobacteriaceae*	Park and Park (2024)
Primary common bile duct stones	Cholangitis without stones	Bile	16S rDNA	Phyla: Proteobacteria, Firmicutes, and Bacteroidetes Genus: *Pseudomonas, Escherichia-Shigella*	Liu et al. (2023))
	Without hepatobiliary diseases	Bile, duodenal fluid	16S rDNA	Phyla: Proteobacteria, Firmicutes Genus: *Pseudomonas, Escherichia-Shigella*	Lyu et al. (2021)
	Gallbladder polyps	Bile	16S rDNA	Phyla: Firmicutes, Proteobacteria, Bacteroidota, and Actinobacteriota Genus: *uminococcus, Blautia, and Enterococcus*	Xiao et al. (2024)
Recurrent common bile duct stones	Primary CBD stones	Bile	16S rDNA	Phyla: Proteobacteria, Firmicutes Genus: *Klebsiella, Escherichia-Shigella*	Tan et al. (2022)
	Non-stone biliary stenosis	Bile	16S rDNA	Phyla: Proteobacteria, Firmicutes Genus: *Escherichia*	Ye et al. (2020)
	Non-recurrence CBD stones	Bile	16S rDNA	Phyla: Proteobacteria, Firmicutes Genus: *Escherichia, Klebsiella, Citrobacter, and Streptococcus*	Choe et al. (2021)
	Healthy controls + Primar stones	Duodenal mucosa, bile, BDS	16S rDNA	Phyla: Proteobacteria, Firmicutes, Synergistetes, Actinobacteria, and Fusobacteria Genus: *Enterococcus, Fusobacterium, Escherichia, and Klebsiella*	Liu et al. (2024)
Pigment stone	biliary obstruction without stones	Bile	16S rDNA	Phyla: Proteobacteria, Firmicutes, Bacteroidetes, Fusobacteria, and Actinobacteria Genus: *Enterobacteriaceae, Pseudomonas, Escherichi, Enterococcus Lactobacillus, and Streptococcus*	Kim et al. (2021)
	Self-control	Bile, stone	16S rDNA	Phyla: Proteobacteria, Firmicutes, Bacteroidota, Synergistota, and Actinobacteriota Genus: *Shigella, Enterococcus, Bacteroides, Pseudomonas, Clostridium sensu stricto 13, and Pyramidobacter*	Zhang et al. (2025)
Giant common bile duct stones	with non-giant CBDS	Bile	16S rDNA	Phyla: Firmicutes Genus: *Enterococcus, Citrobacter, Lactobacillus, Pyramidobacter, Bifidobacterium, and Shewanella*	Dai et al. (2023)
Multiple stones	single stone	Bile	16S rDNA	Phyla: Proteobacteria Genus: *Klebsiella, Aquabacterium, Morganella, and Diaphorobacter*	Xia et al. (2025)

## Methodological heterogeneity and its impact on conclusions

Methodological differences among studies may lead to inconsistencies in the conclusions of biliary microbiota studies. Both the selection of sample collection method and sequencing technology may bias the reporting of microbiota composition.

Bile obtained by endoscopic retrograde cholangiopancreatography may become contaminated with duodenal fluid during sample collection. Some studies have found that *Stomatobaculum, Lachnoanerobaculum, Atopobium*, and *Oribacterium*, those belonged to oral cavity taxa (Cai et al., 2023) and dominant duodenal bacteria such as *Bacteroides* and *Clostridium* can often be detected in ERCP-derived bile samples, and these bacteria may not really colonize the biliary tract (Lyu et al., 2021). In some studies, duodenal fluid is also collected as a contrast sample to rule out retrograde contamination and explore the true source of biliary microbiota. At present, 16S rRNA gene amplification and sequencing and shotgun metagenomic sequencing are mainly used in this field. 16S sequencing has the limitations of insufficient resolution, lack of functional information, bias, and inability to distinguish between live and dead bacteria, which may cause biased results (Brooks et al., 2015; de Souza et al., 2025). Metagenomic sequencing can achieve species and strain level identification, and directly detect functional genes and drug resistance genes (Vanni, 2021). However, the concentration of bacterial DNA in bile is extremely low (Fierer et al., 2025), and the technology requires a high starting amount of DNA, which limits the application of large-scale cohort studies. Therefore, 16sRNA is still used for most biliary microbial sequencing.

## The influence of host factors on the biliary microbiota of common bile duct stones

### History of endoscopic sphincterotomy

Endoscopic sphincterotomy (EST) is the standard treatment for common bile duct stones. However, this procedure may result in damage of function of the sphincter of Oddi. Gut microbes may enter the bile ducts, thereby altering the composition of the biliary microbiota. In patients who relapsed after EST, the abundance of *Pyramidobacter* was higher than in patients without a history of EST (Shen et al., 2020). A Another study found a significant increase in biliary microbiota in patients with sphincter of Oddi dysfunction (Liu et al., 2022). These findings suggest that dysfunction of the sphincter of Oddi may alter the bile flora. At the same time, the bacterial community in the patient's biliary tract will also shift to a bacterial community more associated with gallstones. The biliary tract environment formed after EST provides an ideal place for the colonization of certain stone-promoting bacteria. There was a significant increase in the abundance of *Bilophila* and *Shewanella algae* in patients with relaxation of sphincter of Oddi after EST (Liang et al., 2016).

### Duodenal diverticula

Early study showed that juxtapapillary duodenal diverticula may lead to ascend infection by bacteria that produce β-glucuronidase (Egawa et al., 2010). This hypothesis mainly speculates that duodenal diverticula may affect the biliary microecology based on anatomical proximity and microbiota function. However, it lacks a comparison of the biliary microbiota in patients without duodenal diverticula. A recent study found that compared with patients without duodenal diverticulum, patients with common bile duct stones with duodenal diverticulum showed significant enrichment of Enterococcus and Klebsiella in bile (Wang et al., 2025). Although the evidence is still insufficient, it has been shown that duodenal diverticulum is associated with changes in biliary microbial composition in patients with common bile duct stones. Therefore, future studies should fully consider this influencing factor in the design to further clarify its clinical significance.

### History of antibiotic use

We usually believe that the use of antibiotics will affect the structure of the microbial community (Szajewska et al., 2025). One study found significant differences in β diversity between patients with and without a history of EST in primary common bile duct stones without antibiotics. However, there was no significant difference in the overall composition of the microbiota after antibiotic treatment (Shen et al., 2020). This study indicates that antibiotics can affect the overall microbial community structure. Many studies considered the history of antibiotic use as an important influencing factor. However, the duration of antibiotic use in the exclusion criteria was inconsistent, ranging from 3 days to 6 months. Future studies should establish standardized exclusion times and routinely conduct comparative analyses with and without antibiotic history to ensure that true microbiota differences are observed.

### Diet

The influence of diet on gut microbiota has been validated in several studies. Studies have shown that a high-fat, low-fiber pro-inflammatory dietary pattern can change the composition of gut microbiota (Bailén et al., 2020), thereby affecting bile acid metabolism and enterohepatic circulation (Deng et al., 2026; Xia et al., 2025). However, there is no evidence from direct studies on whether and how diet directly affects the biliary microbiota in patients with common bile duct stones. The current evidence mainly comes from studies on the effect of diet on the microbiota of gallstones. A study found that dairy intake was negatively associated with the abundance of *Bacteroidobacteria* in the gallbladder microbiota (Gutiérrez-Díaz et al., 2018). Given the similarities in the pathogenesis of common bile duct stones and gallstones, as well as the close relationship between intestinal microbiota and biliary microbiota, it is speculated that diet may also have a regulatory effect on biliary microbiota in patients with common bile duct stones. However, this hypothesis needs to be tested in studies specifically targeting the common bile duct stones population.

## Microbial mechanisms associated with common bile duct stones

Studies have suggested that a variety of biliary microorganisms may be involved in the biological pathways of common bile duct stone formation. However, the majority of biliary microbiota studies of common bile duct stones have been categorized at the C1 level (observed association). Although these studies are unable to establish causality, they help provide key clues for specific biomarkers of different subtype of common bile duct stones.

### Biofilm formation and heterogeneous nucleation

Biofilm formation is a widely conserved bacterial persistence mechanism characterized by adhesion to surfaces followed by the formation of a protective extracellular matrix. Biofilm formation as an alternative bacterial mechanism underlying pigment stone formation was originally proposed by Stewart et al. They suggested that bacteria form biofilms by secreting extracellular polysaccharides, which may provide heterogeneous nucleation sites for stones (Sutherland, 2001). Swidsinski et al. found that neither the gallbladder wall nor the bile duct had biofilms. Bacteria detected in biliary stents, calcified pancreatic ducts, or gallstones may use foreign bodies or destructive anatomical changes to form biofilms (Xia et al., 2025). One study also found that bacterial imprinting and bacterial biofilms on the surfaces of the stones *via* scanning electron microscopy, where bacteria were encapsulated in aggregates, forming abundant extracellular biofilms (Cheng et al., 2016). Another study based on metagenomics found that genes involved in biofilm formation were identified in *Enterococci* isolated from pigment stones (Kose et al., 2018). The discovery of biofilm-related genes in biliary tract bacteria has elevated the evidence from simple morphological observation to genetic mechanisms, but the causal relationship remains unconfirmed.

### Bacterial slime and mucin

In addition to the role of biofilms, researchers also believe that extracellular polymers (EPS) secreted by bacteria are closely related to stone formation. In a study involving 370 patients, Stewart et al. (2000) found that 73% of infected pigment stones contained EPS-producing bacteria, whereas β-glucuronidase was produced in only 38% (Stewart et al., 2000). This suggests that the correlation between EPS and stone formation may be stronger than the traditional enzymological view. However, these conclusions are mainly from cross-sectional studies, and it is not possible to distinguish whether EPS is a contributing factor to stone formation or a secondary result of cholestasis or the presence of foreign bodies. In addition, host-derived mucin has also attracted attention. Bacterial infection can promote mucin expression, and mucin, as a matrix and scaffold for stone growth, may accelerate stone formation (Zhao et al., 2024). This hypothesis in causal validation is still lacking. Existing studies mostly focus on gallstones, and future studies need to further verify its specific contribution to common bile duct stones.

### Lipopolysaccharide (LPS)

Lipopolysaccharide (LPS), an endotoxin of Gram-negative bacteria, can promote stone formation through a variety of pathways. As early as 1997, (Osnes et al. 1997) found that the concentration of LPS in bile was significantly increased in patients with common bile duct stones and that LPS was an independent predictor of stone formation (Osnes et al., 1997). Hao et al. (2022) performed metagenome sequencing in patients with choledocholithiasis complicated with cholangitis, and found that the KEGG pathways related to LPS biosynthesis in the intestinal flora were significantly altered (Hao et al., 2022). This can provide metagenomic evidence for the role of LPS in the pathogenesis of choledocholithiasis. Yu et al. (2024) revealed an immunological mechanism by which LPS promotes stone formation by inducing NETs (Yu et al., 2024). Although the conclusion mainly comes from gallbladder stones, this study provides reference for future research ideas and experimental methods to explore the causal relationship between common bile duct stones.

### β-glucuronidase

β-glucuronidase was the first bacterial enzyme that was found to be involved in gallstone formation. As early as 1966, Maki proposed that β-glucuronidase produced by bacteria could hydrolyze conjugated bilirubin to free bilirubin, and then free bilirubin combined with calcium ions to form insoluble bilirubin calcium, which was then precipitated as the core of bile pigment stones (Maki, 1966). Osnes et al. analyzed the bile of 86 patients with choledocholithiasis and found that β-glucuronidase activity was also significantly increased in the bile of patients with choledocholithiasis (Osnes et al., 1997). Subsequent studies have further enriched this mechanism. Skar et al. (1988) identified *Escherichia coli, Bacteroides* and *Clostridium* perfringens as the major β-glucuronidase producing strains (Skar et al., 1988). Among them, Leung et al. (2001) found that *C*. perfringens produced 34 times more enzyme activity than E. coli (Leung et al., 2001). This finding suggests that previous studies may have underestimated the role of anaerobic bacteria in biliary tract infection and stone formation. Zheng et al. further found that *Escherichia coli, Enterococcus, Klebsiella, Aeruginosa, Streptococcus, and Staphylococcus* exhibiting β-glucuronidase activity were more common in patients with recurrent common bile duct stones after endoscopic treatment (Choe et al., 2021). Another study revealed that the biliary microbiota of patients with recurrent common bile duct stones exhibits an enhanced capacity for glucuronide and glucuronate degradation, suggesting elevated β-glucuronidase activity (Liu et al., 2024). At the molecular level, Swidsinski et al. used Polymerase chain reaction (PCR) technology to directly detect the uidA gene encoding β-glucuronidase in common bile duct stones, providing genetic evidence for the direct involvement of bacterial enzymes in stone formation (Lee et al., 1999). A recent study also identified species carrying udA, pldA, and plc genes, which encode β-glucuronidase, in the bile of pigmented stones (Zhang et al., 2025). The above evidence is still mainly based on enzymatic analysis and genetic detection, and there is still a lack of causal verification such as sterile animal models or gene knockouts. In addition, whether the relative contribution of β-glucuronidase in different types of stones (common bile duct stones, gallbladder stones, and pigment stones) is different is still inconclusive. Therefore, more causal verification studies are still needed in the future.

### Phospholipase

Phospholipases are another bacterial enzyme thought to be involved in gallstone formation. Study reported that palmitic acid generated by phospholipase hydrolysis of phospholipids in infected bile combines with calcium ions in bile to form water-insoluble calcium palmitic acid precipitates, which participate in the formation of brown pigment stone (Robins et al., 1982). As early as 2015, Shen et al. first identified the gene encoding phospholipase C in biliary tract samples by metagenomic sequencing of bile from gallstone patients, providing direct functional gene evidence for the involvement of phospholipase in stone formation (Shen et al., 2015). However, the study did not further clarify which specific species carry the gene. Based on these findings, Zhang et al. conducted a more in-depth analysis of bile and stone samples from patients with pigment stones. Compared with Shen et al.'s study, Zhang et al. (2025) study not only expanded the detection scope, but also detected two types of phospholipase genes, pldA(encoding phospholipase A1/A2) and plc (encoding phospholipase C), and also identified the specific strains carrying these genes (Zhang et al., 2025).

### Short-chain fatty acid metabolism

Short-chain fatty acids (SCFAs) are the main microbial metabolites of dietary ferritin (Macfarlane and Macfarlane, 2003). The most common SCFAs in the gut are acetate, propionate, and butyrate (Cummings et al., 1987). Enterococcus is the main genus of biliary microorganisms and can metabolize citrate (Sarantinopoulos et al., 2001). Therefore, Enterococcus can metabolize short-chain fatty acids. Many studies have shown that the occurrence of diseases, such as inflammatory bowel disease, type 2 diabetes, and asthma, is related to a decrease in the proportion of “good bacteria” that can produce SCFAs in the gut (Frank et al., 2007; Zaiss et al., 2015; Zhao et al., 2018). SCFAs may play a role in patients with common bile duct stones because of the enrichment of Enterococcus in the bile of patients with common bile duct stones. A recent study revealed significantly elevated levels of acetate and formate in the bile of the common bile duct stones (Lee et al., 2023). SCFAs may reduce the proportion of good bacteria in patients with common bile duct stones. Xiao et al. (2024) reported that the abundance of SCFA-producing actinomycetes was reduced in the common bile duct stone group, leading to impairment of the biliary immune barrier and regulation of inflammation, thus creating a microenvironment conducive to stone formation (Xiao et al., 2024). One study also found that acetate and formate play a role in the metabolism of bile acid and the regulation of cholesterol concentrations. Acetate may regulate bile acid synthesis by activating FXR and indirectly affecting cholesterol solubility (Lavelle and Sokol, 2020). These studies have shown that SCFAs are strongly related to the gut microbiota and that SCFAs may offer insights into pathogenic bacterial proportions and could inform future therapeutic strategies, though clinical validation is needed.

### Bile acid metabolism disorder

**Figure 2:**
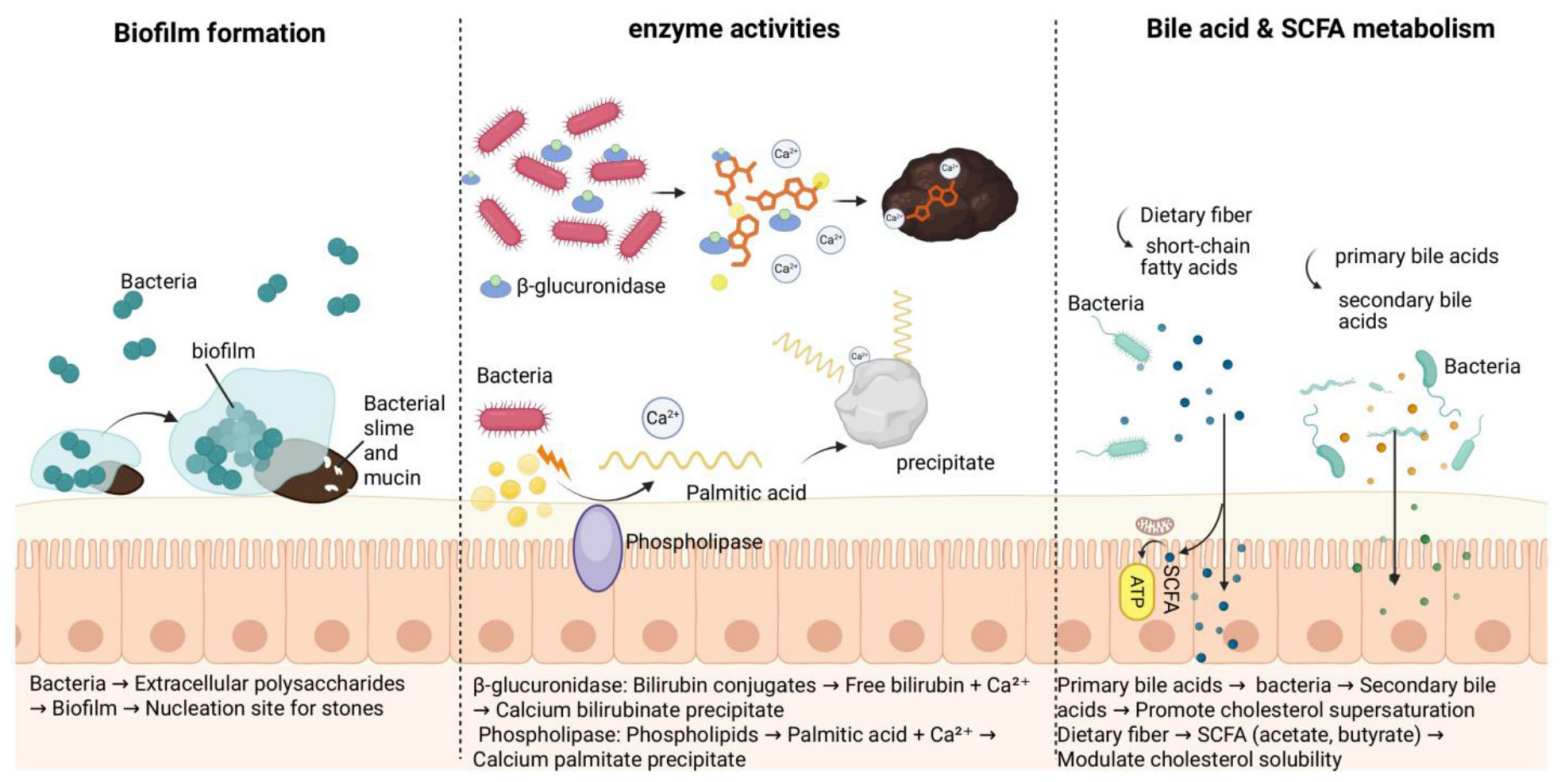
Mechanisms of biliary microbiota-driven stone formation. Left: Biofilm formation provides nucleation sites. Middle: Bacterial enzymes (β-glucuronidase, phospholipase) drive bilirubin and calcium precipitation. Right: Bile acid and SCFA metabolism imbalances modulate cholesterol solubility. Evidence remains associative; causal validation is needed.

Abnormal bile acid metabolism in patients with common bile duct stones manifests as increased binding of BAs, an imbalance in the hydrophobic/hydrophilic ratio, decreased secondary BA production and enterohepatic circulation disorder. These changes together drive the formation and recurrence of common bile duct stones by promoting cholesterol supersaturation, bile duct injury, and inflammation. In a retrospective study, changes in the characteristics of bile acid in patients with common bile duct stones were revealed (Guan et al., 2023). In a study based on a population with giant common bile duct stones, researchers reported abnormal bile acid metabolism, with significant enrichment of free bile acids in the group with giant common bile duct stones and a decreasing trend of combined bile acid abundance (Dai et al., 2023). Deoxycholic acid can reduce the solubility of bile cholesterol and promote the precipitation of cholesterol crystals. No difference in the biliary deoxycholic acid contents is found, but the bile acid content was significantly lower in gallstone patients than in gallstone-free patients. Deoxycholic acid does not cause gallstone formation in patients with cholesterol gallstones (Gustafsson et al., 2000). Steroid lipid-related compounds can combine with calcium ions to produce saponified deposits, leading to the development of stones. Ursodeoxycholic acid is generally believed to cause a common reaction related to lipid metabolism and amino acid metabolism, which can reduce cholesterol and endotoxin levels in bile, inhibit intestinal cholesterol absorption, and ultimately promote gallstone dissolution (Figure 2; Chang et al., 2018; Guan et al., 2022).

## Clinical significance of the biliary microbiota in patients with common bile duct stones

### Potential diagnostic value

Diagnostic tools for common bile duct stones include abdominal ultrasonography, computed tomography, magnetic resonance cholangiopancreatography, and endoscopic ultrasound (Suzuki et al., 2022; Takenaka and Kudo, 2022). These methods have a good effect on the detection of stones, but it is difficult to determine the specific type of stones (such as primary, recurrent, single or multiple, giant stones, etc.). In recent years, preliminary studies have revealed an association between the specific composition of bile microorganisms and different subtypes of common bile duct stones, but these findings are currently observational results, and their diagnostic value needs to be rigorously verified. Several studies have reported differences in microbial composition in different common bile duct stone types. The abundance of Actinobacteria in primary common bile duct stones was significantly reduced (Xiao et al., 2024), and the abundance of *Klebsiella* and *E. coli* in patients with recurrent common bile duct stones was significantly increased (Liu et al., 2024). The unique composition of giant common bile duct stones was mainly composed of *Enterococcus, Citrobacter, Lactobacillus, Bifidobacterium*, and *Shewanella* (Dai et al., 2023). Different types of common bile duct stones have different microbial compositions, which helps identify the type of stone. In a new study on the number of common bile duct stones, *Morganella* was found only in patients with multiple common bile duct stones (Xia et al., 2025). However, all these associations are derived from limited observational studies and have not been validated in prospective cohorts or independent samples. Therefore, these hypothetical diagnostic applications still need to be confirmed by large, well-designed studies.

### Recurrence predictive value

Although ERCP combined with endoscopic sphincterotomy is the main treatment option for common bile duct stones, and the biliary clearance rate is as high as 95%, 25% of patients will develop recurrent bile duct stones after surgery (Mansour et al., 2022). The recurrence of common bile duct stones may be asymptomatic or symptomatic, mainly manifested as obstructive jaundice, acute suppurative ascending cholangitis, pancreatitis, etc. (Ostrow, 1984). To date, no method has been proven to effectively predict the recurrence of common bile duct stones. Currently available findings regarding potential predictors of recurrence are derived from exploratory observational studies. Tan et al. (2022) reported that *Klebsiella* was more abundant in the bile of patients with recurrent common bile duct stones than in that of patients with primary gallstones (Tan et al., 2022). They suggested that the detection of *Klebsiella* may be a potentially effective method for monitoring recurrent common bile duct stones, but this observation has not been validated and does not yet support clinical use. *Enterococci* produce large amounts of β-glucuronidase and are involved in the formation of brown gallstones. Xiao and colleagues suggested that abnormal changes in *Enterococcus* abundance might be a potential marker for predicting the development of common bile duct stones, but this hypothesis awaits testing (Xiao et al., 2024). Although there is no validated marker for predicting recurrence, these preliminary association findings point to several directions for future research. If microbial markers such as *Klebsiella* or *Enterococcus* species can be validated in prospective, large, independent cohorts, they may eventually serve as noninvasive or minimally invasive adjunctive tools for risk stratification of recurrent common bile duct stones.

### Potential therapeutic approaches for common bile duct stones

At present, endoscopic stone removal is the preferred treatment method, among which endoscopic cholangiopancreatography and endoscopic sphincterotomy are the most common (Wu et al., 2021). Although surgical treatment resolved the mechanical obstruction, it did not change the underlying pathogenic environment leading to stone formation. However, it still faces the challenges of high complication rate and high long-term recurrence rate (Disario et al., 2004; Yoon et al., 2023; Zhao et al., 2013). Therefore, microbial therapy has been proposed as a potential complementary strategy. One study found that *Lactobacillus* targeting inhibits cholesterol gallstone formation in a mouse model of cholesterol gallstones (Oh et al., 2021). Another study found a significant protective effect of probiotic intervention against cholesterol gallstone formation induced by high-fat diet (Wang et al., 2025). At present, only studies have found the effect of probiotics on cholesterol stones, and there is a lack of research on the regulation of probiotics on biliary flora in common bile duct stones. Therefore, it is necessary to further establish a common bile duct stones specific model and verify the actual effect of probiotics on the disease.

## Summary and outlook on biliary microorganisms in the diagnosis and treatment of common bile duct stones

With the development of high-throughput sequencing and bioinformatics technology, biliary tract microorganisms have begun to attract people's attention. Current studies have found the association between biliary flora imbalance and common bile duct stones, and proposed a variety of possible mechanisms, including biofilm formation, bacterial enzymes, bile acid metabolism disorders, and short-chain fatty acid imbalance. This study reviewed the different types of common bile duct stones and found that there were specific bacterial flora and microbial composition signatures between different stones. In the future, it is necessary to develop biomarkers for accurate diagnosis and typing on the basis of specific microbial profiles of different common bile stone types and to construct recurrence risk prediction models that integrate microbiome characteristics.

Existing studies have mostly been based on small-sample cross-sectional designs. It is difficult to completely exclude the confounding effects introduced by factors such as underlying diseases and the medication history of patients, resulting in high heterogeneity in the results. The mechanism by which biliary microbiota promotes the formation of common bile duct stones as it is currently understood is mostly based on correlation analysis, and there is a lack of evidence to support causality.

Therefore, longitudinal human intervention and in-depth analysis of the mechanism are needed to address the key question of causality. In view of the microbial spectrum and metabolic characteristics of bile, research should focus on microbial interventions, such as the application of probiotics and fecal microbiota transplantation.
